# β-Elemene suppresses tumor growth of diffuse large B-cell lymphoma through regulating lncRNA HULC-mediated apoptotic pathway

**DOI:** 10.1042/BSR20190804

**Published:** 2020-02-11

**Authors:** Tonglin Hu, Yu Gao

**Affiliations:** 1 Department of Hematology, Zhejiang Provincial Hospital of TCM, The First Affiliated Hospital of Zhejiang Chinese Medical University, Hangzhou, Zhejiang 310006, PR China; 2 Department of Hematology, Zhejiang Hospital, Hangzhou, Zhejiang 310013, PR China

**Keywords:** β-elemene, Apoptosis, DLBCL, HULC, lncRNA

## Abstract

Background: Diffuse large B-cell lymphoma (DLBCL) is considered the most common aggressive subtype of lymphoma. A number of DLBCL patients fail to achieve a response to currently available therapies or develop resistance. β-Elemene is derived from herb Curcuma wenyujin, and exhibits anti-tumor activity in both solid and non-solid tumors through modulating several molecular signaling pathways. We aimed to explore the role of β-elemene in DLBCL treatment and elucidate the involved mechanism.

Materials and methods: Cell viability, apoptosis and expressions of related proteins were assessed and *in vivo* study were performed to determine the tumor suppressive effect of β-elemene and explore the molecular mechanisms.

Results: β-Elemene significantly suppressed the viability of DLBCL cells, and β-elemene down-regulated the lncRNA HULC expression and regulated key pro-apoptotic and anti-apoptotic proteins to induce significant apoptosis of DLBCL cells. HULC overexpression could decrease the β-elemene induced apoptosis, while HULC knockdown increased the apoptosis in DLBCL cells. *In vivo* study further confirmed that β-elemene could suppress the growth of DLBCL xenograft and regulate the HULC expression and the critical proteins of the apoptotic pathway.

Conclusion: β-Elemene performs as a tumor suppressor and modulator of HULC-mediated apoptotic pathway in DLBCL and will be an alternative candidate for clinical application.

## Introduction

It is known that diffuse large B-cell lymphoma (DLBCL) is regarded as the most common aggressive subtype of non-Hodgkin lymphoma (NHL). DLBCL represents the leading cause of disease-specific mortality and accounts for approximately 40% of all newly diagnosed NHL cases [1]. DLBCL patients have a variable clinical response to conventional therapeutic treatments, ranging from cure to failure, refractory disease or death. Although the cure rate of localized DLBCL is high, the cure rate of advanced disease is still less than 50% [2]. Recently, advances in novel therapeutic regimens, such as combined chemoimmunotherapy, stem cell transplantation, have greatly improved the overall survival of DLBCL patients. However, 30–40% of DLBCL patients fail to achieve a response to currently available therapies or develop resistance [3]. Therefore, more effective therapeutic agents based on novel targets and oncogenic signaling pathways are necessarily needed.

Recently, numerous active anti-tumor compounds derived from natural herbs have been identified and developed for clinical application. Natural herbs derived compounds show pleiotropic biological activities including anti-carcinogenic activity and fewer side effects [4–12]. β-Elemene (C_15_H_24_) is derived from herb Curcuma wenyujin, and exhibits a wide spectrum of biological functions including anti-microbial, anti-inflammatory, anti-atherosclerosis and anti-tumor. Previous studies have shown that β-elemene exerted anti-proliferative in both solid and non-solid tumors through modulating several molecular signaling pathways [13–15]. However, the effects of β-elemene on treating lymphoma, especially against DLBCL, and the possible molecular mechanisms remain unelucidated.

Long non-coding RNA (lncRNA) that is greater than 200 nucleotides has been reported to be associated with the development of lymphoma, including DLBCL [16–18]. Aberrant expression of lncRNA is associated with DLBCL occurrence and development. Highly up-regulated in liver cancer (HULC), located on chromosome 6p24.3, is a 500 nt-nucleotide-long lncRNA which is first found overexpressed in liver cancer and is closely related to the tumor progression. HULC is also aberrantly up-regulated in a wide spectrum of malignancies, including liver cancer, prostate cancer, ovarian cancer, bladder cancer, osteosarcoma, gastric cancer, and myeloid leukemia [19–25]. A meta-analysis indicated that HULC overexpression is a predictor of poor prognosis in various cancer types and the higher incidence of tumor metastasis [26]. Up-regulated HULC promotes cellular proliferation, migration, and invasion, while silencing of HULC inhibits tumor growth and enhances chemotherapy-induced apoptosis [24,25]. HULC predicted poor clinical outcome and represented pro-oncogenic activity in DLBCL [27]. Therefore, HULC could serve as a therapeutic target for DLBCL treatment.

In the present study, the antitumor effect of β-elemene in DLBCL was investigated and the potential mechanism through regulation of lncRNA HULC-mediated apoptotic pathway was examined. Our study might provide a new insight in the DLBCL treatment.

## Materials and methods

### Chemicals

**Figure 1:**
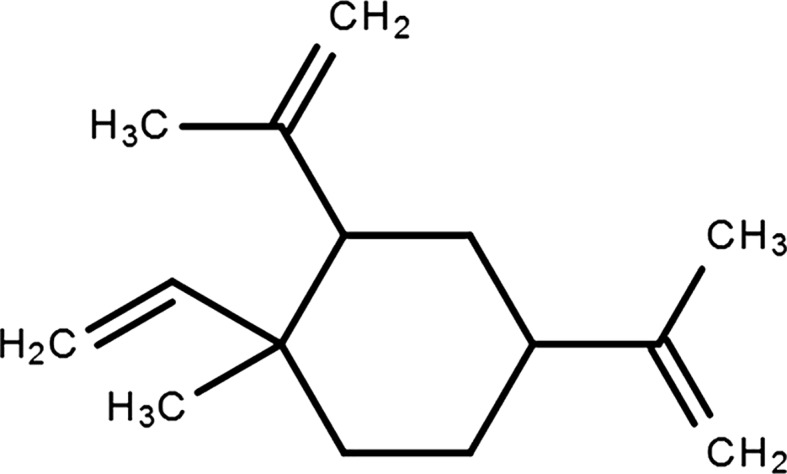
The structural formula of β-elemene

β-Elemene (Figure 1) was purchased from Holley Kingkong Pharmaceutical Co. (Dalian, China). Fetal bovine serum was purchased from Gibco (MA, U.S.A.). RPMI-1640 medium was purchased from Hyclon (UT, U.S.A.). Annexin V-FITC apoptosis kit was purchased from Abcam (MA, U.S.A.). Bax, Bcl-2 antibodies and GAPDH were purchased from Santa Cruz Biotechnology (CA, U.S.A.).

### Cell lines

Human diffuse large B-cell lymphoma cell lines SU-DHL-8, SU-DHL-10 were purchased from American Type Culture Collection (VA, U.S.A.). Cells were maintained in RPMI-1640 medium supplemented with 10% fetal bovine serum at 37°C in a humidified incubator containing 5% CO_2_.

### Cell proliferative inhibition and clonogenic assay

The cytotoxicity of β-elemene on DLBCL cells was measured by MTT assay. Cells (1 × 10^4^ cells/well) were seeded into 96-well plates overnight and then exposed to various concentrations of β-elemene for 12, 24, 48 h. The microplate reader was used to measure the absorbance at 550 nm wavelength and calculate the cell viability rates.

Cells (1 × 10^3^ cells/well) were seeded into 6-well plates and cultured overnight, then exposed to increasing concentrations of β-elemene for 48 h. Cells were cultured in RPMI-1640 medium for further 2 weeks. At the end of the incubation, the colonies were stained and scored under an optical inverted microscope.

### Cell apoptosis analysis

The apoptotic rate of DLBCL cells induced by β-elemene was measured by the Annexin-V/PI apoptosis assay. Cells (5 × 10^4^ cells/well) were seeded into 6-well plates overnight and then exposed to various concentrations of β-elemene for 48 h. Cells were stained with FITC and PI according to the manufacturer’s protocol. Apoptotic cells were calculated as either FITC+/PI− staining or FITC+/PI+ staining by flow cytometry.

### Quantitative real-time transcription-polymerase chain reaction (qRT-PCR)

Total RNAs were extracted from cells by Trizol reagent. qRT-PCR was performed and all kits were used following the manufacturer’s protocol. The primes of HULC: forward, 5′-ACCTCCAGAACTGTGATCCAAAATG-3′, reverse, 5′-TCTTGCTTGATGCTTTGGTCTG-3′. β-Actin was used as endogenous control.

### Cell transfection

HULC full-length cDNA was amplified using PCR and HULC was knocked down using a specific hairpin shRNA. The shRNA sequences for HULC were 5′-GAAGTAAAGGCCGGAATATTC-3′(shHULC-1), 5′-GGAATTGGAGCCTTTACAAGG-3′(shHULC-2). Then, the objective products were cloned into a lentiviral plasmid and infected into cells according to the manufacturer’s protocol.

### Western blot analysis

Cells were exposed to β-elemene (60 μg/ml) for 48 h and washed with PBS and lysed in a RIPA buffer. The lysates were centrifuged (10,000 rpm, 10 min, 4°C) and the supernatant was collected. Equivalent amounts of protein were loaded and resolved on 10% sodium dodecyl sulfate-polyacrylamide gel electrophoresis. Then, the protein was transferred onto a polyvinylidene fluoride membrane which was blocked with TBS-T containing 5% defatted milk. The proteins on the membranes were incubated with Bax, Bcl-2 primary antibodies (1:1000) at 4°C overnight, and then incubated with HRP-conjugated secondary antibody (1:2000) at room temperature for 60 min. The enhanced chemiluminescence system was used to visualize the bands.

### Nude mice xenograft study

The animal experiment was performed at Zhejiang Hospital following the NIH guidelines. The protocol was approved by the ethics committee of Zhejiang Hospital (Hangzhou, China). Male BALB/C mice (4-week-old) were purchased from Shanghai SLAC Laboratory Animal Co. (Shanghai, China). The establishment of DLBCL xenograft was carried out according to the protocol previously described [28,29]. Briefly, the SU-DHL-8 cells (1 × 10^7^ cells) were planted into the upper flank region of mice subcutaneously. Tumor sizes were calculated by the formula (width)^2^ × length/2. When the average tumor size reached 0.2 cm^3^, β-elemene (45 mg/kg) was injected into nude mice intraperitoneally once a day. Saline was used in the control group accordingly. Body weight of nude mice and tumor volume were scored every week. Finally, mice were killed by cervical dislocation at day 28 and tumor samples were collected.

### Immunohistochemistry

Immunohistochemistry was used to determine the expression of Bax and Bcl-2. Paraffin slides (4 μm-thick) were deparaffinized and rehydrated. The slides were under 98°C, 10 min for antigen retrieval prior to incubation with endogenous peroxidase blocking solution. The slides were then incubated with Bax and Bcl-2 antibodies at 4°C overnight, and then incubated with secondary antibody for 1 h. DAB substrate was used and all slides were counterstained with hematoxylin for 4 min. The number of immunopositive cells was identified as positive cells (200× magnification).

### Statistical analysis

The data were presented as mean ± SD from triplicated experiments. Statistical analyses were performed using SPSS version 17.0 (SPSS Inc., Chicago, IL, U.S.A.). One way analysis of variance and SNK-q test were used to assess the differences between groups. Statistical significantce was considered as *p* < 0.05.

## Results

### β-Elemene suppressed the viability of DLBCL cells

**Figure 2:**
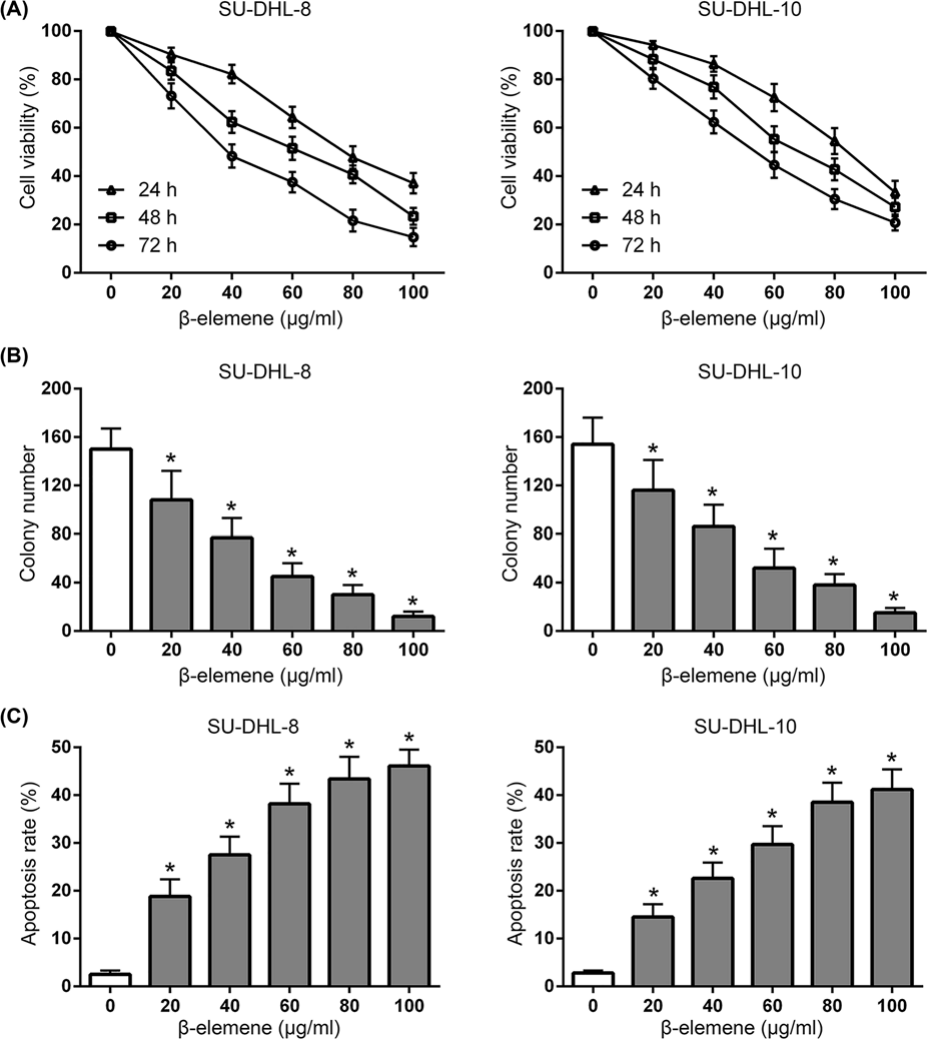
Antitumor effect of β-elemene on the viability of DLBCL cells (**A**) β-Elemene significantly inhibited the viability of SU-DHL-8 and SU-DHL-10 cells in a dose- and time-dependent manner. (**B**) β-Elemene significantly inhibited the colony formation of SU-DHL-8 and SU-DHL-10 cells in a dose-dependent manner at 48 h. (**C**) β-Elemene significantly induced the apoptosis of SU-DHL-8 and SU-DHL-10 cells in a dose-dependent manner at 48 h. *P < 0.05 between β-elemene and control group.

Cell viability of SU-DHL-8 and SU-DHL-10 cells exposed to β-elemene was determined by MTT assay. It showed that β-elemene suppressed the viability of SU-DHL-8 and SU-DHL-10 cells in a dose- and time-dependent manner (Figure 2A). Clonogenic assay also showed that the colony formation ability of SU-DHL-8 and SU-DHL-10 cells were inhibited by β-elemene (Figure 2B). The results showed the anti-proliferative effect of β-elemene on DLBCL cells.

### β-Elemene triggered apoptosis of DLBCL cells

The apoptosis of SU-DHL-8 and SU-DHL-10 cells exposed to β-elemene was further evaluated. The results showed that β-elemene triggered the apoptosis of SU-DHL-8 and SU-DHL-10 cells in a dose-dependent manner (Figure 2C). These data indicated that β-elemene triggered significant apoptosis of DLBCL cells through an apoptotic pathway.

### β-Elemene down-regulated HULC and activated apoptotic pathway

**Figure 3:**
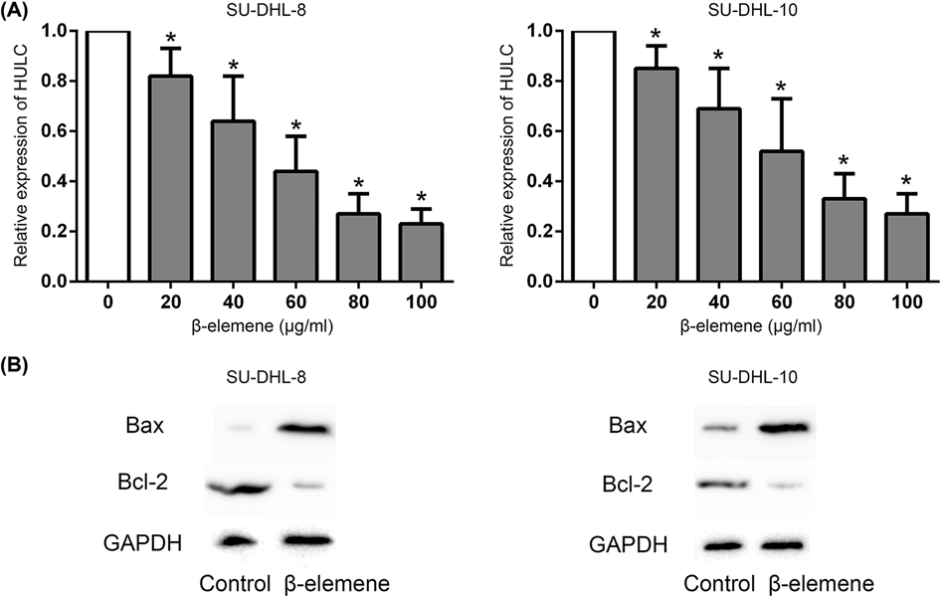
β-Elemene down-regulated HULC and activated apoptotic pathway in DLBCL cells (**A**) β-Elemene significantly down-regulated the HULC expression in a dose-dependent manner. *P < 0.05 between β-elemene and control group. (**B**) β-Elemene (60 μg/ml) up-regulated Bax expression, while down-regulated Bcl-2 expression in SU-DHL-8 and SU-DHL-10 cells at 48 h.

We further investigated the effect of β-elemene on HULC and key pro-apoptotic and anti-apoptotic proteins in SU-DHL-8 and SU-DHL-10 cells. It showed that β-elemene significantly down-regulated the HULC expression in a dose-dependent manner at 48 h (Figure 3A). The result of Western blot analysis showed the up-regulated Bax expression and down-regulated Bcl-2 expression by β-elemene in SU-DHL-8 and SU-DHL-10 cells at 48 h (Figure 3B).

**Figure 4:**
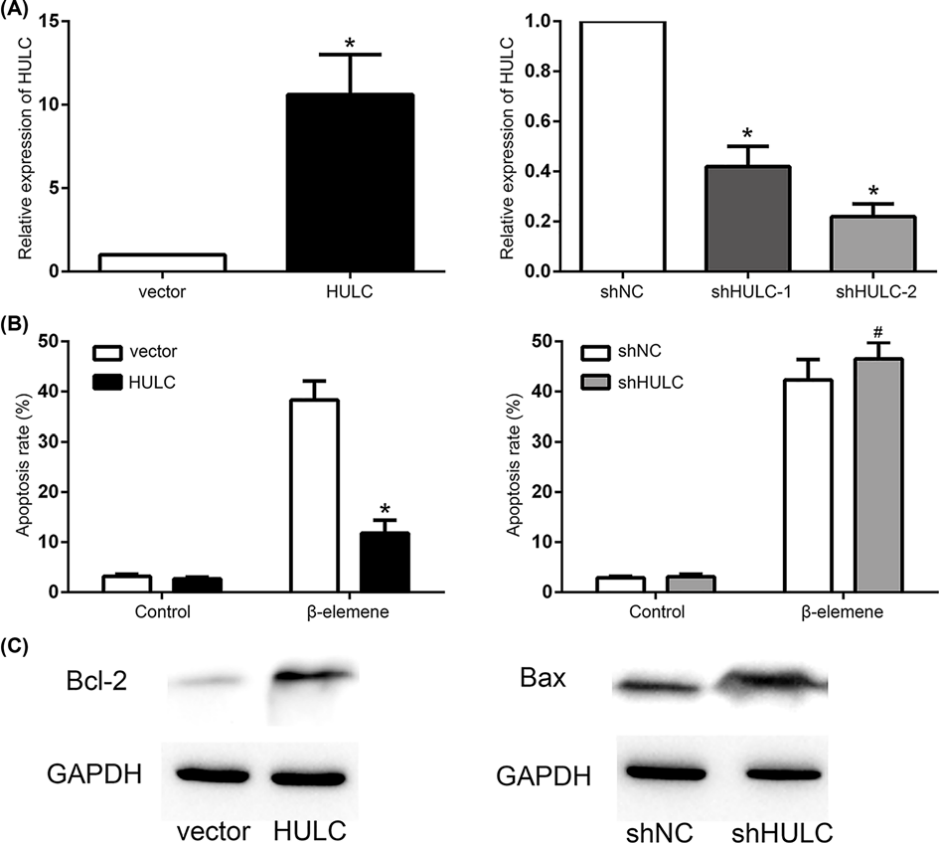
β-Elemene induced apoptosis through HULC-mediated apoptotic pathway in DLBCL cells (**A**) HULC was up-regulated after transfection and down-regulated by knockdown of HULC in SU-DHL-8 cells. *P < 0.05 between β-elemene and control group. (**B**) Apoptosis rate was decreased in HULC overexpression SU-DHL-8 cells treated by β-elemene (60 μg/ml) and no significant change was found in HULC knockdown SU-DHL-8 cells treated by β-elemene (60 μg/ml) at 48 h. *P < 0.05 between HULC and vector group. #*P* > 0.05 between shHULC and shNC group. (**C**) The expression of Bax decreased, while Bcl-2 increased in HULC overexpression SU-DHL-8 cells treated by β-elemene (60 μg/ml), and the expression of Bax increased, while Bcl-2 decreased in HULC knockdown SU-DHL-8 cells treated by β-elemene (60 μg/ml) at 48 h.

To explore the role of HULC in β-elemene induced apoptosis in DLBCL cells, SU-DHL-8 cells were infected with lentivirus to stably overexpress HULC or knockdown of HULC (Figure 4A). The results showed that the apoptosis rate decreased in SU-DHL-8 cells with HULC overexpression, while no significant change was found in the cells with HULC knockdown, which were exposed to β-elemene (60 μg/ml) at 48 h (Figure 4B). The Bax expression decreased, while Bcl-2 expression increased in the β-elemene (60 μg/ml) treated SU-DHL-8 cells with HULC overexpression, and the Bax expression increased, while Bcl-2 expression decreased in the β-elemene (60 μg/ml) treated HULC knockdown SU-DHL-8 cells (Figure 4C).

### β-Elemene suppressed the growth of DLBCL xenograft through regulating HULC-mediated apoptotic pathway *in vivo*

**Figure 5:**
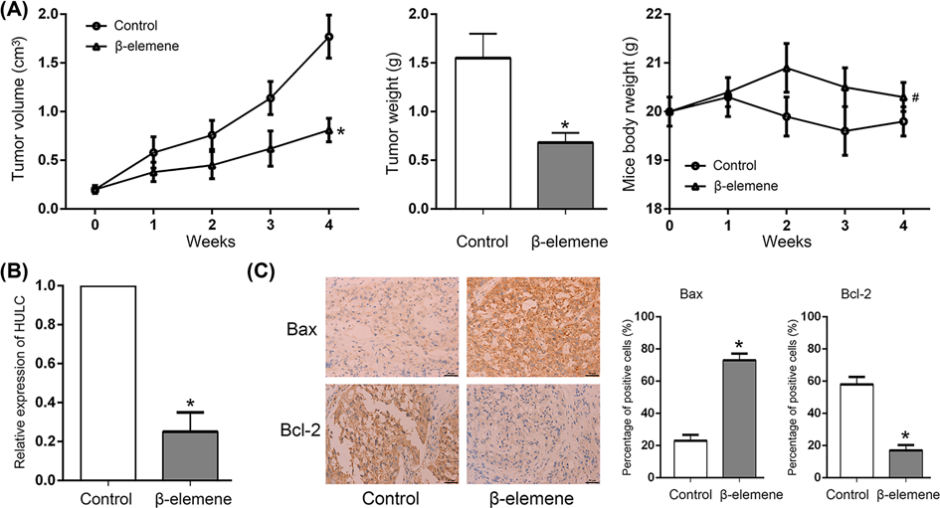
β-Elemene inhibited tumor growth of DLBCL xenograft (**A**) β-Elemene inhibited tumor volume and weight of SU-DHL-8 xenograft without mice body weight changed. *P<0.05 between β-elemene and control group. #*P* > 0.05 between β-elemene and control group. (**B**) β-Elemene down-regulated the HULC expression *in vivo*. *P<0.05 between β-elemene and control group. (**C**) Immunohistochemical analysis showed β-elemene increased Bax expression, while decreased Bcl-2 expression *in vivo*. *P < 0.05 between β-elemene and control group.

SU-DHL-8 xenograft was established to evaluate the antitumor effect of β-elemene *in vivo*. It showed that β-elemene suppressed the tumor growth of SU-DHL-8 xenograft without mice body weight changed (Figure 5A). qRT-PCR showed that the expression of HULC significantly reduced (Figure 5B), and immunohistochemical analysis showed that the Bax expression increased, while Bcl-2 expression decreased by β-elemene treatment (Figure 5C).

## Discussion

Cancer research and recent studies implicated that various lncRNAs are dysregulated in human malignancies. Aberrant expression of lncRNA is associated with the DLBCL patient’s clinical characteristics and poor prognosis and can be applied to serve as a therapeutic target [16–18]. LncRNA HULC has been reported to play a crucial role in tumor carcinogenesis and progression. High expression of HULC was positively associated with advanced malignancies and poor survival of patients. HULC promotes tumor growth and progression in several cancers by mediating multiple signaling pathways and interacting with miRNAs. It has been indicated that HULC accelerated liver cancer by inhibiting PTEN via autophagy cooperation to miR15a and increasing HMGA2 expression via sequestration of the miR186 [19,30]. HULC modulated the phosphorylation of YB-1 through serving as a scaffold of extracellular signal-regulated kinase and YB-1 to enhance hepatocarcinogenesis [31]. HULC promoted proliferation and osteogenic differentiation of bone mesenchymal stem cells via down-regulation of miR-195 [32]. HULC mediated radioresistance via autophagy in prostate cancer cells [33]. HULC functioned as an oncogene by targeting ATG7 and ITGB1 in epithelial ovarian carcinoma [21]. HULC promoted bladder cancer cells proliferation but inhibited apoptosis via regulation of ZIC2 and PI3K/AKT signaling pathway [22].

Therefore, lncRNA HULC may be a therapeutic target for cancer therapy. Knockdown of HULC could inhibit proliferation, migration, invasion, and promote apoptosis by sponging miR-122 in osteosarcoma [23]. Silencing of HULC enhanced chemotherapy-induced apoptosis in gastric cancer [24]. HULC silencing suppressed angiogenesis by regulating ESM-1 via the PI3K/Akt/mTOR signaling pathway in human gliomas [34]. Recently, a growing number of novel treatment strategies, such as compounds derived from natural products, have been developed for cancer therapy by targeting lncRNAs [35–37]. Previous study reported that β-elemene inhibited the growth of esophageal cancer by modulating lncRNA-mediated inhibition of hTERT [38]. However, the role of HULC in β-elemene induced apoptosis in DLBCL remains unclear.

In the present study, we evaluated the antitumor effect of β-elemene and preliminarily elucidated the HULC involved mechanism. The results revealed that β-elemene significantly suppressed the viability of DLBCL cells in a dose- and time-dependent manner. β-Elemene down-regulated the lncRNA HULC expression and regulated key pro-apoptotic and anti-apoptotic proteins to induce significant apoptosis of DLBCL cells. HULC overexpression could decrease the β-elemene induced apoptosis, while HULC knockdown increased the β-elemene induced apoptosis in DLBCL cells. *In vivo* study further confirmed the antitumor effect of β-elemene in the DLBCL xenograft, and the HULC expression and the critical proteins of the apoptotic pathway were regulated by β-elemene. These results suggested that HULC-mediated apoptotic pathway was essential for β-elemene to inhibit DLBCL progression.

In summary, our study indicated that the antitumor activity of β-elemene in DLBCL was attributed to the inhibition of cell viability, induction of apoptosis and regulation of HULC-mediated apoptotic pathway. LncRNA HULC may be a potential therapeutic target for β-elemene in treating DLBCL, and β-elemene will be an alternative candidate for clinical application in DLBCL patients. However, further investigations of the deep mechanisms involved in HULC-mediated apoptosis induced by β-elemene are needed.

## Abbreviations

DLBCL: diffuse large B-cell lymphoma
lncRNA: long non-coding RNA
NHL: non-Hodgkin lymphoma
qRT-PCR: quantitative real-time transcription-polymerase chain reaction
